# Novel Technique of Surgical Management of Scimitar Syndrome

**DOI:** 10.1155/2019/6932680

**Published:** 2019-05-06

**Authors:** Mary Lark, Amanda Cai, Phillip Rideout, David Gregg, Pal Suranyi, Fred A. Crawford, Valerian L. Fernandes

**Affiliations:** ^1^College of Medicine, Medical University of South Carolina, Charleston, SC 29425, USA; ^2^Department of Cardiology, Medical University of South Carolina, Charleston, SC 29425, USA; ^3^Department of Radiology, Medical University of South Carolina, Charleston, SC 29425, USA; ^4^Department of Surgery, Medical University of South Carolina, Charleston, SC 29425, USA

## Abstract

Scimitar syndrome is a rare congenital abnormality resulting from right-sided pulmonary venous return to the inferior vena cava rather than to the left atrium. It is usually detected in early childhood with symptoms of recurrent chest infection and finding of pulmonary hypertension due to left to right shunt. We report a case of a 40-year-old woman with scimitar syndrome discovered on chest X-ray during evaluation of recurrent pneumonia. Surgical correction was achieved with a novel technique of using a synthetic graft connecting the scimitar vein across the right atrium to the left atrium along with ligation of the scimitar vein connection to the inferior vena cava. The patient continues to do well 10 years after surgery, and the shunt graft shows good flow on echocardiogram. We present her clinical and imaging data and details of the surgical technique along with a brief review of surgical literature.

## 1. Introduction

**Figure 1:**
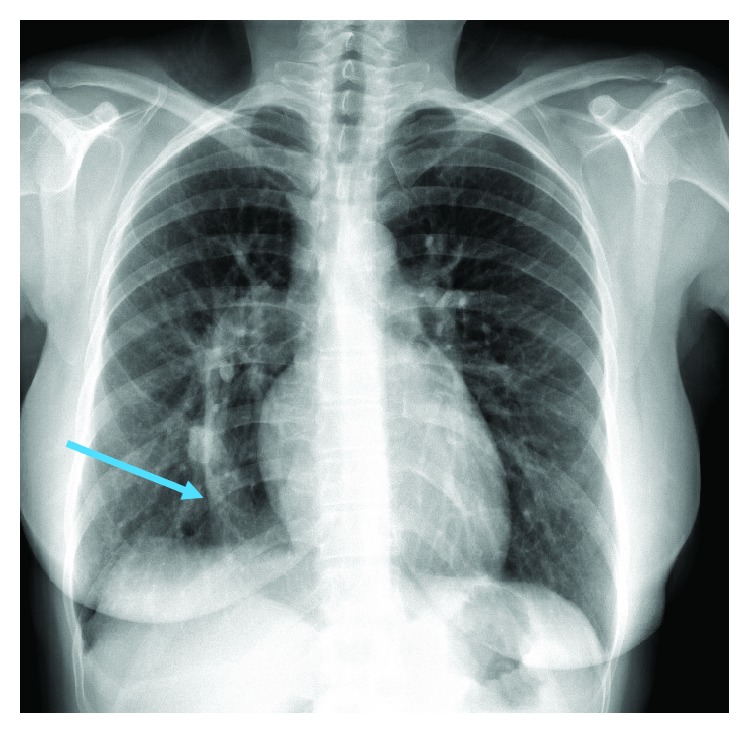
Frontal upright PA CXR of patient. Arrow pointing to scimitar vein in the right lower lung.

Scimitar syndrome is a variant of partial anomalous pulmonary venous connection (PAPVC), which results in pulmonary venous connection from the right lung to the inferior vena cava (IVC) [1]. The name is derived from a curvilinear (scimitar-shaped) shadow alongside the right heart border on chest X-ray (Figure 1). Scimitar syndrome is usually associated with other cardiac and pulmonary defects, including atrial septal defects (ASD), dextroposition of the heart, right lung hypoplasia, and pulmonary hypertension [2]. Severe cases in childhood result in cyanosis and pulmonary hypertension, requiring surgical correction. However, some cases are not discovered until adulthood, either due to worsening, symptomatic left to right shunt, or incidental discovery on chest X-ray after recurrent pulmonary infections. Surgical repair of these defects is especially difficult, with several methods requiring cardiopulmonary bypass to repair this anomaly. We present a novel surgical technique used in the repair of scimitar syndrome in a middle-aged woman.

## 2. Case Report

A 40-year-old Caucasian woman presented to her primary physician with recurrent fevers, chills, wheezing, and cough. She was a welder by profession and had a long history of respiratory symptoms and allergies. She was treated with a course of antibiotics for a presumed pneumonia. When her cough and other symptoms persisted, a chest X-ray was performed and showed a curvilinear abnormality alongside her right heart border consistent with scimitar syndrome (Figure 1). Upon further review of history, the patient endorsed worsening shortness of breath over the past year and occasional palpitations with exertion, but denied symptoms with normal activity. She had recurrent pulmonary infections and asthma/allergy episodes throughout childhood and adult life. Physical exam showed normal jugular venous pressure (JVP). She had a parasternal heave but her apex was not localized. The heart sounds were normal and she did not have any murmurs.

Echocardiogram showed dilatation of the right atrium and right ventricle with and interatrial shunt consistent with a patent foramen ovale (PFO). Ejection fraction was normal.

**Figure 2:**
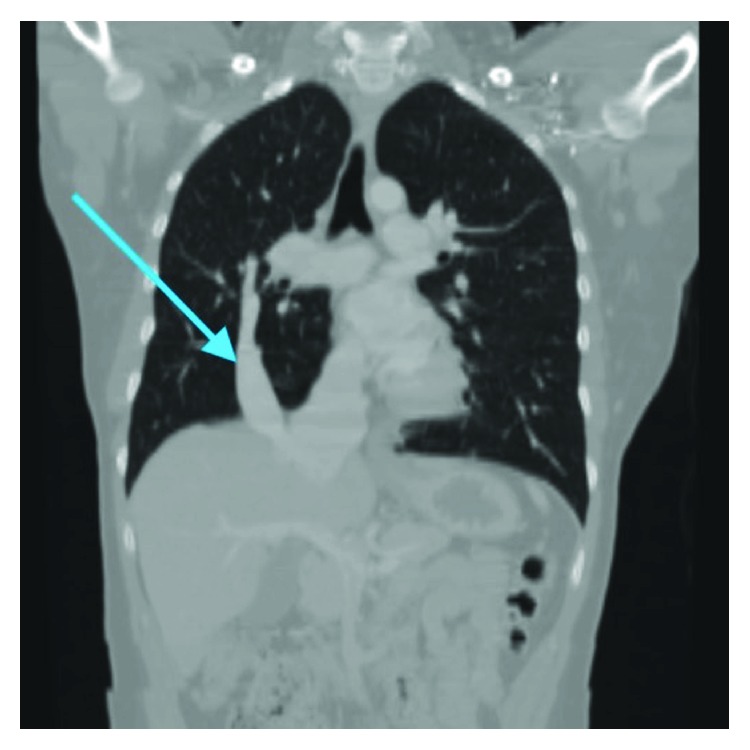
Coronal CT with IV contrast. Arrow pointing to scimitar vein in the right lower lobe, which drains into the IVC.

Computed tomography (CT) of the chest (Figure 2) showed a hypoplastic right upper lobe and compensatory large right lower lobe. There was a large right pulmonary artery with a large arteriovenous malformation (AVM) from the pulmonary artery into the anomalous scimitar vein which attached to the inferior vena cava. The majority of the right lung pulmonary venous return was to the inferior vena cava via the scimitar vein. A single small vestigial inferior pulmonary vein on the right extended to the left atrium. The left pulmonary veins drained normally into the left atrium.

Transthoracic echocardiography (TEE) confirmed right atrial and right ventricular dilatation and normal left pulmonary venous return into the left atrium. There was an atretic right inferior pulmonary vein opening into the right atrium. The right lower pulmonary vein was not visible by echocardiography. A PFO was confirmed by color Doppler and agitated saline injection.

**Figure 3:**
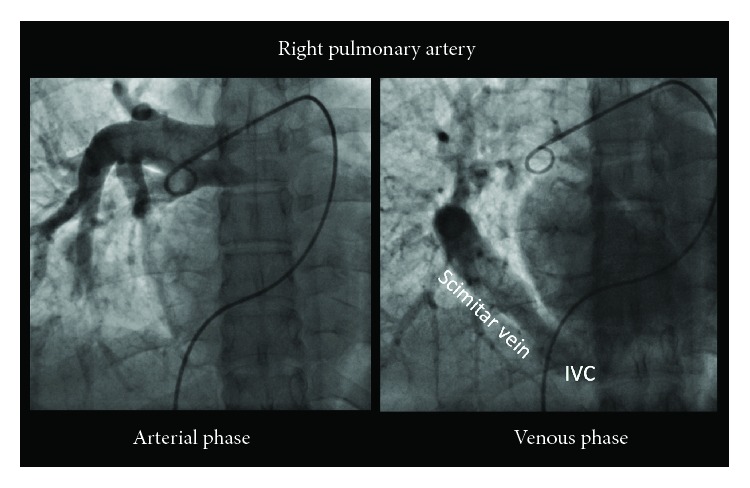
Pulmonary angiogram in anterior-posterior projection. The pigtail catheter is in the right pulmonary artery. The arterial phase [left frame] reveals the right pulmonary arteriogram. The right frame shows the venous phase with the scimitar vein from the right upper lobe draining into the IVC.

Cardiac catheterization showed normal coronary arteries and normal left ventricular end-diastolic pressure. The PFO allowed easy passage of the multipurpose catheter into the left atrium. Pulmonary angiography, aortogram, and scimitar venography were also performed using a 6F pigtail catheter. Right pulmonary artery angiogram identified partial anomalous pulmonary venous return via scimitar vein to the IVC (Figure 3). The patient was referred for surgical correction due to persistent shunt and recurrent pulmonary infections. Upon multidisciplinary review of the case, the traditional approach of reimplanting the anomalous scimitar vein into the left atrium was deemed to carry excessive risk and was therefore deferred. After review of the patient's CT imaging, a simplified interatrial baffle redirecting the anomalous pulmonary venous blood to the left atrium was pursued.

## 3. Surgical Technique

**Figure 4:**
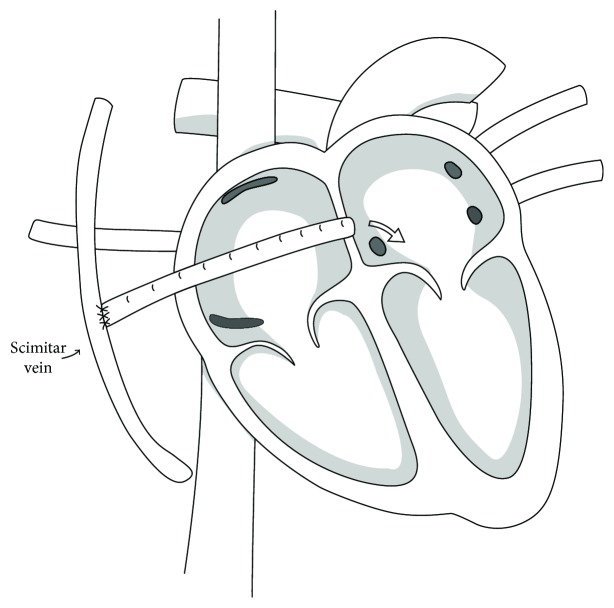
Schematic diagram of the scimitar vein and the surgically created baffle. The baffle connects the scimitar vein to the left atrium via the right atrium. The blue arrow points to the ligated scimitar vein.

After satisfactory general anesthesia, the patient was placed on cardiopulmonary bypass. With the heart completely decompressed, the lung was retracted. The anomalous vein was identified in an oblique fissure of the right lung and demonstrated to drain through the diaphragm. The anomalous vein was dissected for a distance of several centimeters from the diaphragm up and toward the hilum of the lung. The right atrium was opened through a longitudinal incision approximately 1.5 cm away from the AV groove. There was a large fossa ovalis and a small patent foramen ovale. These landmarks were used as guide for excising the entire fossa ovalis to a diameter of approximately 1.6 to 1.8 cm. A ribbed 16 mm Gore-Tex graft was chosen and sutured end-to-side to this newly created atrial septal defect with continuous 5-0 Prolene suture. A linear incision slightly smaller than the graft was made very low in the lateral right atrial wall, and the graft was brought through this defect and then through a defect in the pleura. A linear incision was made in the anomalous pulmonary vein, and the graft was sutured to this vein meticulously with a continuous 5-0 Prolene suture (Figure 4). After confirming both anastomosis and the patency of the graft, the anomalous vein was ligated between the Gore-Tex graft and the diaphragm. The patient tolerated the procedure well and was discharged on warfarin four days after the procedure.

## 4. Postoperative

**Figure 5:**
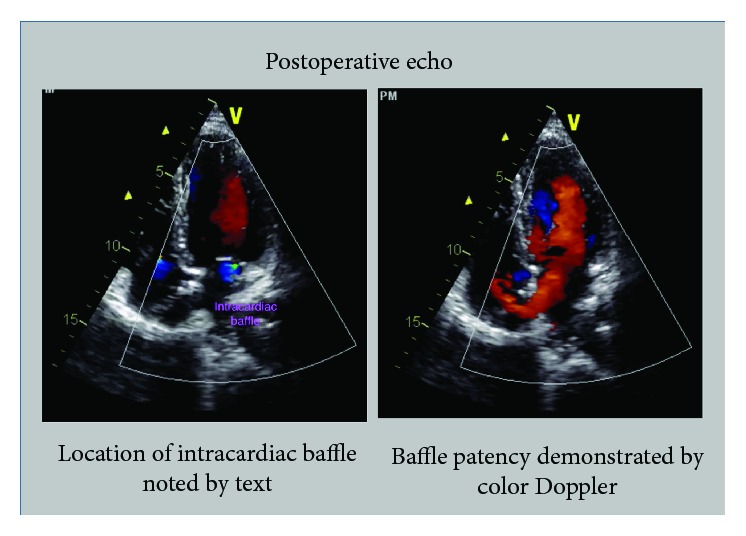
Echocardiogram, apical four chamber view. The left frame shows the baffle traversing the right atrium and opening into the left atrium. The right frame shows color flow through the baffle into the left atrium.

**Figure 6:**
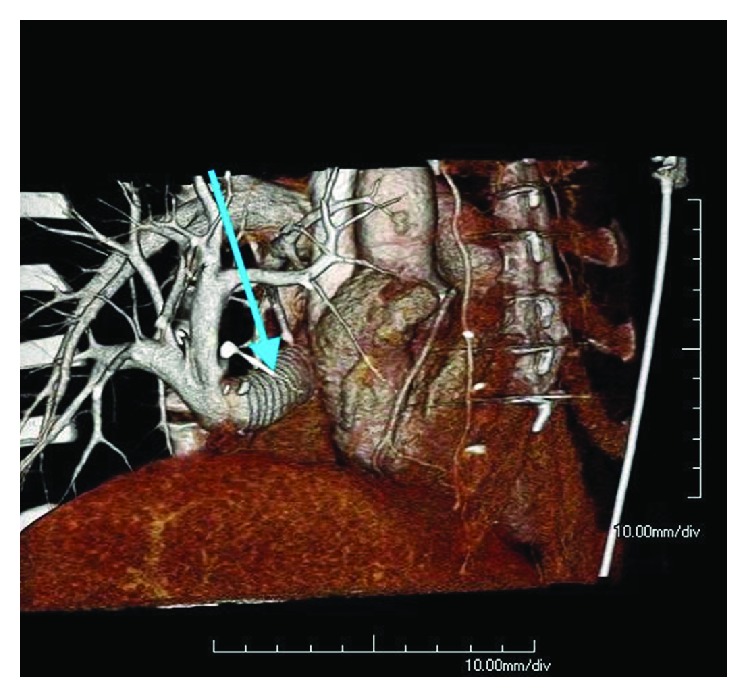
3D volume rendering of postoperative CT angiogram from a right anterior oblique view. Arrow pointing to the artificial baffle, draining the scimitar vein, aiming posteriorly toward the left atrium.

**Figure 7:**
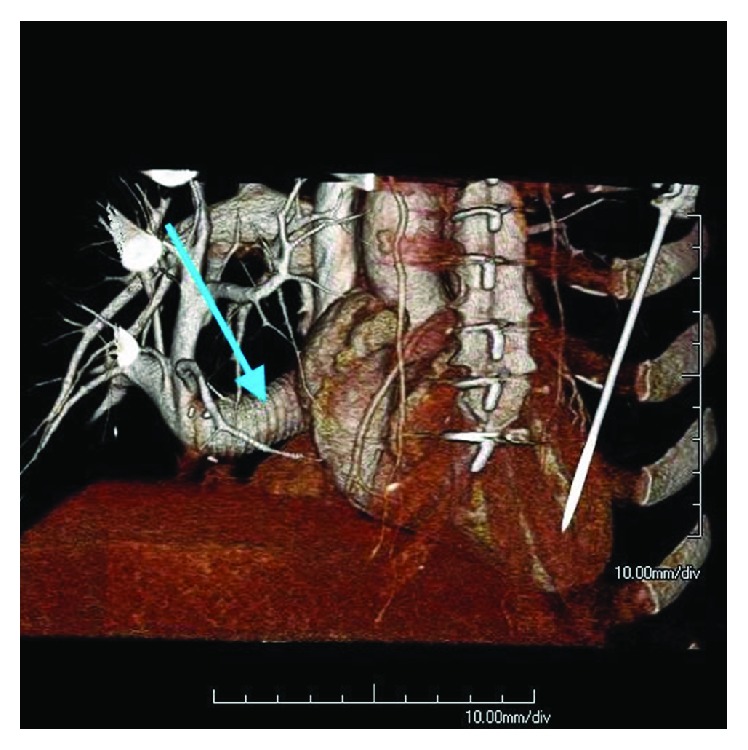
3D volume rendering of postoperative CTA from a less oblique RAO view. Arrow pointing to the artificial baffle diverting the scimitar vein toward the left atrium behind the right atrium.

**Figure 8:**
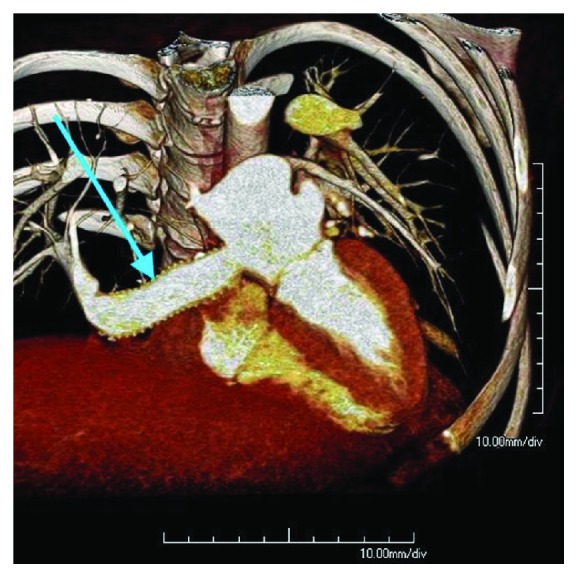
3D volume rendering, cutaway view, of postoperative CTA. Arrow pointing to the artificial baffle connecting the scimitar vein with the left atrium via the posterior aspect of the right atrium.

**Figure 9:**
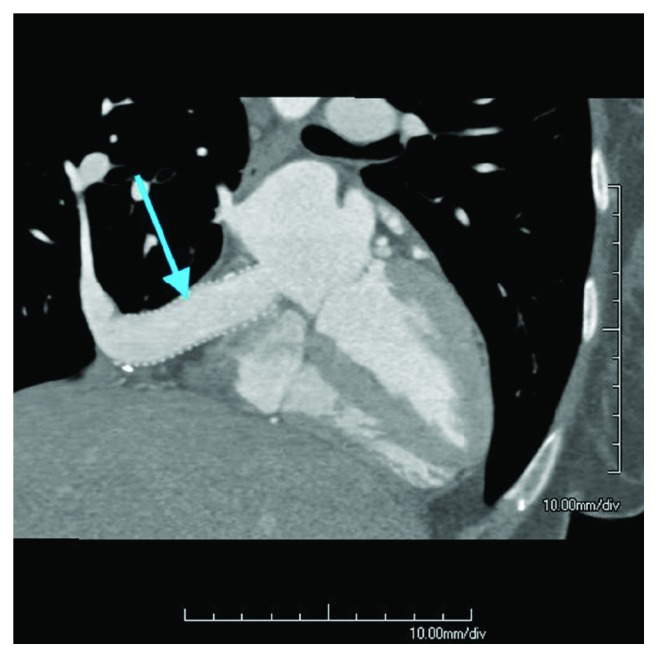
Oblique multiplanar reformat of postoperative CTA. Arrow pointing to the widely patent artificial baffle connecting the scimitar vein with the left atrium via the posterior aspect of the right atrium. There is no filling defect inside the graft to suggest thrombus.

ECHO showed a right ventricular peak systolic pressure of 22 mmHg with no intracardiac shunts. The graft passing through the right atrium was noted to be patent with good flow on Doppler (Figure 5). A pulmonary perfusion scan was performed which showed a differential perfusion of 47.8% to the left lung and 52.2% to the right lung. One-month postoperative cardiac-gated CTA showed that the synthetic graft remained widely patent with no evidence of thrombus (Figures 6–9).

The patient noted an uneventful recovery with less fatigue and improvement in her breathing during follow-up visits. She has continued to do well for 10 years after the procedure. The shunt was well-visualized with good flow on serial follow-up echocardiograms.

## 5. Discussion

Surgical correction of scimitar syndrome in adults is usually reserved for symptomatic patients with worsening left to right shunt. Numerous surgical techniques have been described, with most requiring deep hypothermia and circulatory arrest. The first corrective operation was performed by Kirklin and associates [3] in 1956 without the use of cardiopulmonary bypass. Shumacker and Judd [4] devised a method using cardiopulmonary bypass, involving direct reimplantation of the anomalous pulmonary vein into the right atrium. This method was modified by Zubiate and Kay [5] in 1962 with the use of an interatrial patch developed to redirect flow into the left atrium through an ASD.

Direct anastomosis of the scimitar vein to the left atrium was first described by Honey [6] in 1977. However, there were frequent complications associated with this method, including inadvertent constriction or occlusion via thrombosis. This thrombosis is attributed to mobilization of the scimitar vein with resultant vessel distortion or the tortuous coursing of the scimitar vein resulting in kinking. Dupuis and colleagues [7] described these complications in a series of 37 patients that underwent classical surgical repair. Of these 37 patients, only 12 had acceptable outcomes without evidence of thrombosis. Additional surgical complications include stenosis of the intracardiac baffle, pulmonary hypertension, and hemoptysis [8].

Our method is based upon the approach described by Harrison and colleagues [9]. We used a Gore-Tex graft penetrating through the walls of the right atrium to connect the scimitar vein to the left atrium via a newly created ASD. This method prevents the complications associated with moving the scimitar vein itself, while successfully correcting the left-to-right shunt through a more direct path than the traditional method. The ease and success of this technique should make it the preferred surgical strategy for surgical repair of symptomatic scimitar syndrome in adults.

## 6. Conclusion

Traditional surgical repair of scimitar syndrome is difficult and complicated. The technique described using synthetic graft baffle provides a more direct path for right-sided pulmonary venous return to the left atrium. The ease and success of this technique and the long-term graft patency should make it the preferred surgical strategy for surgical repair of symptomatic scimitar syndrome in adults.
